# Pediatric dilated cardiomyopathy and the progression to heart failure: mechanisms and interventions focusing on the “gene-inflammation-metabolism” axis

**DOI:** 10.3389/fcvm.2026.1888470

**Published:** 2026-08-07

**Authors:** Jinsong Jiang, Dong Luo, Yan Gu, Ziyu Wang, Yanyan Xiao, Aihua Hu

**Affiliations:** 1 Department of Non-Communicable Disease Management, Beijing Children’s Hospital, Capital Medical University, National Center for Children’s Health, Beijing, China; 2 Department of Cardiology, Beijing Children’s Hospital, Capital Medical University, National Center for Children’s Health, Beijing, China

**Keywords:** genetics, heart failure, inflammation, metabolism, pediatric dilated cardiomyopathy, precision medicine

## Abstract

Dilated cardiomyopathy (DCM) is the most common cardiomyopathy in children and a leading indication for heart transplantation. Despite considerable progress in understanding its genetic architecture, translating these insights into improved outcomes for children remains challenging. The pathophysiology of pediatric dilated cardiomyopathy is highly complex, extending beyond a simple monogenic model to encompass intricate interactions between genetic predisposition, inflammatory triggers, and metabolic dysregulation. This review systematically synthesizes current evidence on the mechanisms driving heart failure progression in pediatric dilated cardiomyopathy, focusing on the integrated framework of the “gene-inflammation-metabolism” axis. We begin by outlining the genetic landscape of pediatric dilated cardiomyopathy, highlighting key pathogenic genes and their associated phenotypes, with particular emphasis on features distinct from adult disease. We then explore how environmental “second hits” (such as viral infection and autoimmune responses) trigger excessive inflammatory reactions, and discuss the significance of special clinical phenomena such as “hot phase” myocarditis in genetically susceptible children. Concurrently, we systematically examine the metabolic reprogramming of the failing heart, including mitochondrial dysfunction, substrate utilization shifts, insulin resistance, and iron metabolism disturbances, analyzing how these processes further compromise energy-starved cardiomyocytes. By integrating these three interconnected domains, this review presents a pediatric-specific triaxial framework that unifies known DCM pathways into a single actionable model for precision risk stratification and targeted therapy. This framework supports the development of novel biomarkers, multi-dimensional risk stratification strategies, and targeted interventions—ranging from anti-inflammatory agents and metabolic modulators to gene therapy, psychological support, and AI-assisted risk prediction. This review aims to provide a novel theoretical framework and practical roadmap for the clinical management and future research of pediatric dilated cardiomyopathy.

## 1 Introduction

Heart failure is a complex clinical syndrome that represents the final common pathway of various cardiovascular diseases (1). In the pediatric population, dilated cardiomyopathy (DCM) is the most common type of cardiomyopathy, accounting for approximately 50% of all childhood cardiomyopathies and a leading cause of heart failure and heart transplantation in children (2–4). Epidemiological data indicate that the annual incidence of pediatric DCM ranges from 0.57 to 1.24 per 100,000 children, with the highest rate in infants under one year of age, reaching 4.2 per 100,000, and these infants often present with more severe clinical manifestations and higher rates of heart failure and mortality (3, 5, 6). Heart failure attributable to DCM imposes a substantial disease burden globally, contributing significantly to pediatric morbidity, mortality, and healthcare resource utilization. However, large-scale population-based epidemiological studies specifically focusing on pediatric DCM remain relatively scarce compared with adult populations. Available data from established pediatric cardiomyopathy registries in the United States, Australia, and Finland suggest that the incidence estimates do not differ substantially across these populations.

Although guideline-directed medical therapy for heart failure with reduced ejection fraction (HFrEF) in adults has significantly improved outcomes (7, 8), the prognosis for children with DCM remains poor. Large pediatric cardiomyopathy registry studies report that approximately 31% of children with DCM die or undergo heart transplantation within one year of diagnosis, and 46% within five years. For those not receiving transplantation, the five-year survival rate is approximately 50%–60% (2, 4, 9). This poor prognosis is closely linked to the unique pathophysiological features of pediatric DCM.

The etiology of pediatric DCM is highly complex and heterogeneous. In addition to known causes such as myocarditis, neuromuscular diseases, and inherited metabolic disorders, pathogenic gene mutations can be detected in approximately 40% of affected children (4, 10, 11). These pathogenic genes are widely distributed across those encoding sarcomeric proteins, cytoskeletal proteins, nuclear envelope proteins, and ion channels, demonstrating significant genetic heterogeneity (12, 13). However, the traditional dichotomy of “genetic” vs. “acquired” DCM no longer adequately explains the complex pathological processes. In recent years, the “two-hit” hypothesis, initially established in adult studies, has gained attention in pediatric DCM, suggesting that genetic predisposition can be unmasked or exacerbated by environmental factors (such as viral infection, autoimmune response, metabolic stress), thereby triggering disease onset and progression (14, 15). Unlike in adults, where “second hits” often include hypertension, coronary artery disease, or chemotherapy, the pediatric “second hits” are more commonly viral myocarditis, autoimmune responses triggered during development, and the metabolic demands of rapid growth and puberty. Furthermore, the developmental stage of the immune and metabolic systems in children may result in distinct inflammatory and metabolic responses to these triggers.

The progression from initial injury to overt heart failure involves complex interactions across multiple molecular pathways. The inflammatory response, as the body's initial defense mechanism against injury, can transform into maladaptive chronic inflammation under certain conditions, driving myocardial fibrosis and ventricular remodeling (16, 17). Concurrently, the failing heart undergoes profound metabolic reprogramming, shifting from fatty acid oxidation-based energy metabolism to less efficient glycolysis, a process exacerbated by mitochondrial dysfunction, leading to energy depletion and further deterioration of systolic function (18, 19). Notably, genetic background, inflammatory response, and metabolic disturbance do not exist in isolation; they interact through complex networks, amplifying each other's effects, and collectively constitute the core mechanism driving the progression from pediatric DCM to heart failure.

Based on this understanding, this review proposes the “gene-inflammation-metabolism” axis as an integrated framework for understanding the mechanisms of heart failure progression in pediatric DCM. We will systematically elaborate on the three core components of this axis and their interactions, synthesize the latest research advances in pediatric DCM, and explore precision medicine strategies based on this framework, aiming to provide new insights for clinical practice and future research.

Literature Search Strategy: This narrative review was conducted following the PRISMA guidelines for scoping reviews. We systematically searched PubMed, Embase, Web of Science, and the Cochrane Library for articles published from database inception to January 2026. The search strategy combined the following keywords and MeSH terms: (“pediatric” OR “child” OR “children” OR “infant” OR “adolescent”) AND (“dilated cardiomyopathy” OR “DCM”) AND (“heart failure” OR “HF” OR “cardiomyopathy”) AND (“genetics” OR “genetic” OR “mutation” OR “variant”) AND (“inflammation” OR “inflammatory” OR “cytokine” OR “immune”) AND (“metabolism” OR “metabolic” OR “mitochondrial” OR “oxidative stress”). Reference lists of included articles and relevant review articles were manually screened for additional eligible studies. We included original research articles, clinical trials, meta-analyses, systematic reviews, and authoritative guidelines published in English. Studies were excluded if they exclusively focused on adult populations without providing mechanistic insights or pediatric implications, were limited to animal models without clinical translation, or lacked sufficient methodological detail. After systematic searching and screening, 159 relevant articles were identified and included in this review.

## 2 The genetic basis of pediatric dilated cardiomyopathy: constructing a susceptible myocardium

Genetic factors are a major cause of pediatric DCM, and understanding the genetic landscape is crucial for elucidating disease mechanisms, guiding clinical management, and predicting prognosis. The genetic architecture of pediatric DCM shares similarities with adult disease but also has unique characteristics.

### 2.1 Genetic epidemiology of pediatric DCM

Familial cases account for approximately 31.6% of pediatric DCM, which is much higher than previously recognized (6, 20). With the widespread application of next-generation sequencing, the diagnostic yield for DCM genetics has significantly increased. Current studies show that approximately 40% of children with DCM have identifiable pathogenic gene variants, a proportion comparable to adult DCM (10, 21). However, due to age-dependent penetrance, some children carrying pathogenic variants may not develop clinical manifestations until adulthood, making genetic diagnosis during childhood more challenging.

The inheritance pattern of pediatric DCM is most commonly autosomal dominant, but autosomal recessive, X-linked, and mitochondrial inheritance also occur (22). Approximately 110 nuclear protein-coding genes and 24 mitochondrial DNA-encoding genes have been associated with DCM, broadly involved in various structural domains and functional modules of cardiomyocytes (23).

### 2.2 Key pathogenic genes and their mechanisms

#### 2.2.1 ***TTN*** gene: the most common cause

The *TTN* gene encodes titin, the largest protein known to be expressed in cardiomyocytes, consisting of approximately 35,000 amino acids that span from the Z-line to halfway through the M-band, forming the core structure of the sarcomere together with thick and thin filaments (24, 25). Titin is not only the “third filament” of the sarcomere but also acts as a molecular spring, providing passive myocardial stiffness and participating in active force regulation (26).

*TTN* truncating variants (TTNtv) are the most common cause of genetic DCM, accounting for approximately 25% of familial cases and 12%–18% of sporadic cases (24, 27), with similar prevalence estimates reported in pediatric cohorts. In pediatric DCM, *TTN* variants are also the most common genetic etiology (21). The primary mechanism by which *TTN* variants cause DCM is haploinsufficiency, where insufficient functional protein is produced from the mutant allele, leading to compromised sarcomeric structural integrity and reduced contractility (28). Induced pluripotent stem cell-derived cardiomyocyte models have confirmed that cardiomyocytes carrying TTNtv exhibit sarcomere deficiency and reduced contractile force (28).

Notably, the carrier frequency of TTNtv in the general population is approximately 1%–3%, much higher than the prevalence of DCM (29). This suggests that TTNtv alone is insufficient to cause DCM and requires additional “second hits” to trigger disease manifestation. This finding provides important clues for understanding gene-environment interactions. Furthermore, the location of *TTN* variants is a key determinant of pathogenicity, with variants located in highly expressed exonic regions (such as the A-band) having higher pathogenicity (30).

In pediatric DCM, *TTN*-associated DCM is typically associated with later age of onset and higher rates of left ventricular reverse remodeling, with a relatively better prognosis (31, 32). This provides important reference for clinical management.

#### 2.2.2 ***LMNA*** gene: a paradigm of malignant phenotype

The *LMNA* gene encodes lamins A and C, key components of the nuclear envelope, produced by alternative splicing (33). Lamins A/C not only maintain the structural integrity of the nuclear envelope but also participate in gene expression regulation, signal transduction, DNA replication, and nucleocytoplasmic transport (34).

*LMNA* mutations account for 5%–10% of genetic DCM cases in combined adult and pediatric cohorts but represent one of the subtypes with the poorest prognosis (33, 35). Clinical features of *LMNA*-DCM include: (1) cardiac conduction system abnormalities, such as atrioventricular block and sinus node dysfunction; (2) high prevalence of atrial and ventricular arrhythmias, with significantly increased risk of ventricular tachycardia and sudden cardiac death; (3) rapid progression of heart failure with poor response to conventional medical therapy (36, 37). Studies show that non-missense mutations (truncating, deletion, insertion) are associated with higher rates of malignant arrhythmias and refractory heart failure compared to missense mutations (38).

The pathogenic mechanisms of *LMNA* mutations involve multiple signaling pathways. In myocardial tissue from *LMNA*-deficient mice, abnormal activation of the mTOR pathway has been observed, and treatment with mTOR inhibitors such as rapamycin can improve cardiac function at the cellular level (39). Additionally, abnormal activation of the MAPK signaling pathway contributes to *LMNA*-DCM development, and inhibitors targeting this pathway (such as ARRY-371797) have shown efficacy in early-phase clinical trials (40). Notably, arrhythmias can precede left ventricular dilation and systolic dysfunction, a feature that aids in early identification of *LMNA*-DCM patients.

Given the severe clinical consequences associated with *LMNA* mutations, the 2019 Heart Rhythm Society guidelines recommend that even with preserved systolic function, consideration should be given to implantable cardioverter-defibrillator implantation for primary prevention of arrhythmias in patients with *LMNA* mutations (41).

#### 2.2.3 Sarcomeric genes: from hypertrophy to dilated phenotype

Sarcomeric gene variants, traditionally associated with hypertrophic cardiomyopathy, can be detected in approximately 5%–10% of DCM patients (42, 43), though pediatric-specific prevalence estimates remain less well-defined. These genes include *MYH7* (encoding *β*-myosin heavy chain), *MYBPC3* (encoding myosin-binding protein C), *TNNT2* (encoding cardiac troponin T), *TNNI3* (encoding cardiac troponin I), and *TPM1* (encoding *α*-tropomyosin).

The mechanism by which sarcomeric gene variants cause DCM differs from hypertrophic cardiomyopathy, primarily manifesting as reduced contractility and impaired energy metabolism. For *MYH7*, mutations can disrupt the normal structure and function of the myosin head, leading to abnormal cross-bridge cycling and reduced contractility (44). *TNNT2* mutations can interfere with troponin-calcium binding, disrupting calcium dynamics and the contraction-relaxation rhythm of cardiomyocytes (45). *MYBPC3* truncating mutations, through haploinsufficiency, increase the proportion of myosin in the active DRX conformation, ultimately leading to excessive contraction, impaired relaxation, and increased energy expenditure (46).

In pediatric DCM, sarcomeric gene variants are associated with early-onset, severe phenotypes, and lower rates of left ventricular reverse remodeling compared to *TTN*-associated DCM (47, 48). Additionally, some sarcomeric gene variants can cause childhood-onset left ventricular non-compaction, overlapping with DCM phenotypes (49).

#### 2.2.4 Other high-risk genes

In addition to the genes mentioned above, several other gene variants are strongly associated with pediatric DCM and often carry a high risk of malignant arrhythmias.

*FLNC* gene: encodes filamin C, an important Z-disc protein involved in actin crosslinking and signal transduction (50). *FLNC* truncating variants can cause arrhythmogenic DCM, with cardiac magnetic resonance often showing a characteristic “ring-like” late gadolinium enhancement pattern (51). Studies report that in DCM cohorts carrying *FLNC* mutations, the incidence of malignant ventricular arrhythmias and sudden cardiac death during five-year follow-up reaches 15%–20% (51). Therefore, consideration should be given to implantable cardioverter-defibrillator implantation for primary prevention in these patients.

*DSP* gene: encodes desmoplakin, a key component of desmosomes that maintains cardiomyocyte intercellular junction integrity (52). *DSP* variants can cause arrhythmogenic cardiomyopathy and DCM, often presenting with “hot phase” myocarditis, characterized by acute myocarditis-like episodes (elevated troponin, arrhythmias, inflammatory changes on cardiac magnetic resonance) (53). Cardiac magnetic resonance often shows a “ring-like” late gadolinium enhancement pattern, suggesting extensive myocardial fibrosis (54).

*RBM20* gene: encodes RNA-binding motif protein 20, which acts as a splicing factor regulating alternative splicing of multiple genes including *TTN* (55). *RBM20* mutations can cause aggressive, early-onset DCM with high rates of ventricular arrhythmias and sudden cardiac death risk (56, 57). Induced pluripotent stem cell-derived cardiomyocyte models have confirmed that *RBM20* mutations affect not only *TTN* splicing but also the splicing of calcium-handling-related genes such as *CAMK2D* (58).

*PLN* gene: encodes phospholamban, which regulates sarcoplasmic reticulum calcium pump activity (59). The *PLN* p.Arg14del founder variant, common in Dutch and German populations, can cause severe DCM/arrhythmogenic cardiomyopathy with high arrhythmic burden and sudden cardiac death risk (60, 61). This variant leads to abnormal distribution of phospholamban on the cell membrane, causing abnormal calcium exchange and membrane potential instability (62).

*BAG3* gene: encodes BCL2-associated athanogene 3, involved in protein homeostasis regulation and autophagy (63). *BAG3* variants are associated with pediatric DCM, with male patients having earlier onset and higher heart failure risk (63, 64).

*SCN5A* gene: encodes the *α*-subunit of the cardiac sodium channel, primarily involved in the rapid depolarization phase of action potentials (65). *SCN5A* variants are associated not only with primary arrhythmias such as long QT syndrome and Brugada syndrome but can also cause DCM (66). DCM patients carrying *SCN5A* mutations often have associated conduction system abnormalities and a high risk of malignant arrhythmias (67). Certain specific mutations (such as *SCN5A*-R222Q) can enhance sodium channel excitability and may be targeted with sodium channel blockers such as lidocaine (67).

Table 1 systematically summarizes key genes associated with pediatric DCM, including their prevalence, key phenotypes, arrhythmic risk, heart failure risk, and key notes, providing reference for clinical genetic counseling and risk assessment.

**Table 1:** Key genes with definitive or strong causal association with pediatric dilated cardiomyopathy.

Gene	Protein	Prevalence in Pediatric DCM	Key Associated Phenotypes	Arrhythmic Risk	HF Risk	Key Notes and References
*TTN*	Titin	15–20% (most common)	DCM, LVNC	Moderate	Moderate	Most common cause; later onset; higher LVRR potential (24, 27, 31)
*LMNA*	Lamin A/C	5–10%	DCM, conduction disease, muscular dystrophy	Very High	Very High	Malignant course; early arrhythmias; high SCD risk; poor LVRR (33, 35, 37)
*MYH7*	*β*-myosin heavy chain	2–5%	DCM, HCM, LVNC	Moderate	High	Can cause early-onset, severe DCM; LVRR lower than *TTN* (42, 47, 48)
*TNNT2*	Cardiac troponin T	1–2%	DCM, HCM	Moderate	Moderate	Sarcomeric DCM; variable penetrance (42, 43)
*FLNC*	Filamin C	1–3%	DCM, ACM	High	Moderate	Arrhythmogenic; ring-like LGE on CMR; high SCD risk (50, 51)
*DSP*	Desmoplakin	1–3%	ACM, DCM	High	Moderate	Fibrotic phenotype; “hot phase” myocarditis; ring-like LGE (52–54)
*RBM20*	RNA-binding motif protein 20	1–2%	DCM, ACM	High	High	Aggressive, early-onset; affects *TTN* splicing; high VT risk (55–57)
*PLN*	Phospholamban	<1%	DCM, ACM	High	High	p.Arg14del founder variant; severe arrhythmic burden; poor prognosis (59–61)
*BAG3*	BCL2-associated athanogene 3	1–2%	DCM	Moderate	High	Earlier onset in males; higher HF risk in males (63, 64)
*SCN5A*	Nav1.5 sodium channel	1–2%	DCM, arrhythmic syndromes	Moderate	Low	Overlap with conduction disease; can present as tachycardia-induced DCM (65–67)

Some prevalence data are derived from adult studies or limited pediatric studies. ACM, arrhythmogenic cardiomyopathy; DCM, dilated cardiomyopathy; HCM, hypertrophic cardiomyopathy; HF, heart failure; LGE, late gadolinium enhancement; LVRR, left ventricular reverse remodeling; LVNC, left ventricular non-compaction; SCD, sudden cardiac death.

### 2.3 Sex differences and gene expression

Recent studies reveal significant sex differences in genetic DCM. In major DCM genetic cohorts, female patients are consistently underrepresented, suggesting lower penetrance of DCM in females (36, 68). Among *BAG3* variant carriers, male sex is associated with earlier age of onset and worse heart failure prognosis, though arrhythmic risk does not differ significantly by sex (32, 63). Among *TTN* and *LMNA* variant carriers, male patients also generally have worse outcomes (37, 69). Furthermore, male sex is a risk factor for ventricular arrhythmias and sudden cardiac death in *LMNA* variant carriers and has been incorporated into indications for implantable cardioverter-defibrillator placement (41).

For *FLNC* variant cardiomyopathy, available evidence suggests a numerically lower risk of major cardiovascular events in women, though larger studies are needed for confirmation (51). A recent UK single-center study showed that women with DCM had higher left ventricular ejection fraction, less left ventricular remodeling, and lower cardiac fibrosis burden at diagnosis, but had higher rates of heart failure events and cardiovascular mortality during the first two years of follow-up (68). These findings suggest that the mechanisms underlying sex differences in heart failure and arrhythmic risk require further investigation.

Opposing roles of androgens and estrogens on cardiac vascular endothelial cells, fibroblasts, and cardiomyocytes have been demonstrated *in vitro* and in animal models, potentially providing clues for understanding sex differences (70).

### 2.4 Clinical implications of genotype-phenotype correlations

Genotype-phenotype correlation studies provide important evidence for clinical management of DCM. Different genotypes are associated with specific clinical features, rates of disease progression, and risks of adverse events, which have important implications for clinical decision-making.

First, genotype can help predict arrhythmic risk. *LMNA*, *FLNC*, *DSP*, *RBM20*, and *PLN* variants are all associated with high risk of ventricular arrhythmias and sudden cardiac death. For patients carrying these variants, enhanced arrhythmia monitoring and consideration of implantable cardioverter-defibrillator implantation for primary prevention are recommended (36, 37, 51, 56, 60). For *LMNA*-DCM in particular, early emergence of conduction system abnormalities or ventricular arrhythmias suggests high risk even when left ventricular ejection fraction is still preserved (71).

Second, genotype can predict response to medical therapy. Studies show significant differences in left ventricular reverse remodeling (LVRR) response to guideline-directed medical therapy across different genotypes. *TTN* variant carriers have the highest LVRR rates, reaching over 50%; sarcomeric gene variant carriers have LVRR rates of approximately 23%–28%; while *LMNA* variant carriers have the lowest LVRR rates (31, 72). These findings have important implications for prognosis assessment and treatment strategy adjustment.

Third, genotype can guide family screening. For probands with identified pathogenic or likely pathogenic variants, family cascade screening should be initiated, with regular follow-up of genotype-positive, phenotype-negative relatives to detect subclinical disease early (73). Studies show that relatives carrying DCM-associated gene variants such as *TTN* and *LMNA* may have subclinical systolic dysfunction (such as reduced global longitudinal strain) even with normal left ventricular ejection fraction (74, 75).

The genetic architecture of pediatric DCM, while sharing many core genes with adult disease, exhibits distinct characteristics in epidemiology, penetrance, phenotype expressivity, and prognosis. To provide a broader perspective on these differences, we summarize the key comparisons between pediatric and adult DCM in Table 2. Collectively, this comparison highlights that pediatric DCM is characterized by early-onset presentation with peak incidence in infancy, a notable proportion of familial cases, and lower transplantation-free survival compared with adult DCM. In terms of management and therapeutic evidence, children remain underserved: heart transplantation is more frequently the only option in pediatric end-stage disease, whereas gene therapy and SGLT2 inhibitor data in children are still nascent, with most evidence derived from ongoing trials or extrapolated from adult studies. These distinctions underscore the urgent need for pediatric-specific investigations.

**Table 2:** Comparison of Key characteristics between pediatric and adult dilated cardiomyopathy.

Feature	Pediatric DCM	Adult DCM	Key References
Annual incidence	0.57–1.24 per 100,000	5–8 per 100,000	(3, 5)
Peak age of onset	Infancy (<1 year) highest risk	Middle age (40–60 years)	(3, 6)
Familial cases	∼31.6%	∼20%–35%	(6, 20)
Most common gene	*TTN* (15%–20%)	*TTN* (15%–25%)	(24, 28)
High-risk genes	*LMNA, FLNC, DSP, RBM20*	*LMNA, FLNC, DSP, RBM20*	(33, 37, 51, 56)
Inflammatory triggers	Viral myocarditis (more common), autoimmune	Viral, autoimmune, toxic	(76, 77, 91)
5-year survival	∼50%–60% (no transplant)	∼70%–80%	(2, 4, 9)
LVRR rate (*TTN*)	>50%	∼50%	(31, 72)
Heart transplantation	Leading indication in children	Common in advanced HF	(2)
Gene therapy trials	Emerging (pediatric-specific trials ongoing)	More advanced	(120, 151, 152)
SGLT2i evidence	Limited, early-phase pediatric studies	Robust, Phase III trials	(126–128)

DCM, dilated cardiomyopathy; HF, heart failure; LVRR, left ventricular reverse remodeling; SGLT2i, sodium-glucose cotransporter 2 inhibitors.

## 3 The inflammatory response: from protection to maladaptation

Inflammation is a key component of the heart's response to injury, playing a dual role in the pathological process of DCM. Against a genetically susceptible myocardial background, moderate inflammation contributes to tissue repair, but excessive or persistent inflammation can drive myocardial remodeling and heart failure progression (16, 76). This section systematically discusses the triggers, molecular mechanisms, and clinical implications of the inflammatory response in pediatric DCM.

### 3.1 Triggers of inflammation

Triggers of the inflammatory response in pediatric DCM are diverse and include several categories.

#### 3.1.1 Viral infection

Viral infection is an important cause of myocarditis and DCM. The most commonly reported cardiotropic viruses in endomyocardial biopsy samples from Europe and the United States include parvovirus B19, enteroviruses (including coxsackievirus groups A and B), adenoviruses, influenza viruses, and human herpesviruses (76, 77). Viral infection can cause myocardial damage through two mechanisms: (1) active replication within cardiomyocytes, directly inducing cardiomyocyte and endothelial cell injury; (2) triggering uncontrolled immune responses through molecular mimicry (78). Incomplete viral clearance and persistent viral genomes can maintain chronic inflammation, promoting progression from myocarditis to ventricular dilation and DCM (79).

However, detection of viral genome does not necessarily indicate pathogenicity. For example, parvovirus B19 has also been detected in non-inflammatory heart disease and healthy hearts, and its presence may represent latent virus rather than replicative activity (80). Currently, more than 500 viral copies per microgram of DNA is considered the threshold for clinical relevance of parvovirus B19; above this threshold, the virus may sustain myocardial inflammation and potentially evolve to a DCM phenotype (81). Assessment of viral transcriptional activity, viral load, infection localization, and patient genetic background helps determine the clinical relevance of infection (77).

#### 3.1.2 Autoimmunity

The role of autoimmunity in DCM pathogenesis has been extensively investigated. Autoantibodies against cardiac-specific antigens, including anti-*β*1-adrenergic receptor antibodies, anti-M2 muscarinic acetylcholine receptor antibodies, anti-cardiac myosin heavy chain antibodies, and anti-cardiac troponin antibodies, can be detected in up to 60% of DCM patients (82–84). Some autoantibodies are thought to play a pathogenic role in DCM or be associated with poor prognosis, arrhythmias, and sudden cardiac death (85–87).

The mechanisms of autoimmune DCM involve complex interactions between genetic susceptibility and environmental factors. In animal models, cardiac myosin can induce myocarditis in genetically predisposed mice (88). In humans, first-degree relatives of DCM patients with positive anti-heart autoantibodies have a higher proportion of asymptomatic left ventricular dilation and a higher five-year rate of progression to DCM (89). Additionally, children with DCM whose mothers have autoimmune disease have higher rates of anti-myocardial antibody positivity (90).

#### 3.1.3 Genetic background-related inflammatory susceptibility

In recent years, the association between genetic background and inflammatory response has gained increasing attention. Patients carrying genetic variants may present with “hot phase” myocarditis, characterized by acute myocarditis-like episodes (53, 91). Autopsy studies have shown inflammatory infiltrates similar to myocarditis in the myocardium of patients who died from arrhythmogenic cardiomyopathy (92). These findings suggest that genetic defects themselves may render the myocardium hypersusceptible to inflammatory triggers, blurring the distinction between “genetic” and “inflammatory” etiologies. Genetic variants associated with DCM or arrhythmogenic cardiomyopathy have also been found in patients with acute and chronic myocarditis (91, 93).

#### 3.1.4 “Hot phase” myocarditis in genetic cardiomyopathies

A particularly distinctive clinical phenomenon in pediatric DCM is the “hot phase” of genetic cardiomyopathies. Patients carrying *DSP* or *FLNC* variants may present with acute myocarditis-like episodes characterized by elevated troponin, ventricular arrhythmias, and inflammatory changes on cardiac magnetic resonance, often as the first manifestation of the underlying genetic disease. The term “hot phase” reflects the active myocardial inflammation that occurs episodically in these patients, resembling acute viral myocarditis but driven by a genetic predisposition.

The clinical importance of recognizing this phenomenon extends beyond diagnosis. First, it enables early genetic testing and family cascade screening, as up to 77% of patients presenting with “hot phase” myocarditis carry pathogenic variants in cardiomyopathy-associated genes, predominantly *DSP* and *FLNC*. Second, it explains the disease trajectory of patients initially diagnosed with “myocarditis” who later develop overt DCM or arrhythmogenic cardiomyopathy features. Third, it has therapeutic implications: immunosuppressive or immunomodulatory therapy may be considered during acute inflammatory flares, though robust evidence is lacking. Autopsy studies further support the inflammatory nature of this process, showing lymphocytic infiltrates in the myocardium of patients with genetic arrhythmogenic cardiomyopathy who died suddenly. The “hot phase” concept thus exemplifies the gene-inflammation axis in its most clinically tangible form and underscores the importance of genetic testing in all children presenting with acute myocarditis.

### 3.2 Molecular mechanisms of the inflammatory response

#### 3.2.1 NLRP3 inflammasome and IL-1 cascade

The NLRP3 inflammasome is a central regulator of the inflammatory response. Damage-associated molecular patterns (such as heat shock proteins, high mobility group box protein 1) released from injured cardiomyocytes and pathogen-associated molecular patterns (such as viral nucleic acids) can activate Toll-like receptors on cardiomyocytes (94). Upon activation, Toll-like receptors induce NLRP3 inflammasome assembly and activation through downstream signaling pathways (such as the MyD88-dependent pathway) (95).

Activated NLRP3 inflammasome cleaves pro-caspase-1 into active caspase-1, which then cleaves pro-IL-1β and pro-IL-18 into mature IL-1β and IL-18 (95). Once released, these pro-inflammatory cytokines directly damage cardiomyocytes while also amplifying the inflammatory response by recruiting and activating immune cells (such as macrophages, neutrophils) (16). This cascade can create a vicious cycle, leading to collagen deposition, fibrotic scar formation, and ultimately promoting ventricular dilation and heart failure progression (95).

#### 3.2.2 Involvement of innate and adaptive immune cells

Both innate immune cells (macrophages, dendritic cells, natural killer cells) and adaptive immune cells (T cells, B cells) participate in the myocardial inflammatory response in DCM (96, 97).

Macrophages are the most abundant immune cell type in the myocardium. Based on expression levels of C-C chemokine receptor 2, myocardial macrophages can be divided into CCR2 + and CCR2- subsets. CCR2 + macrophages are involved in inflammatory responses and fibrosis, while CCR2- macrophages mediate tissue repair (97). In DCM, CCR2 + macrophages recruit monocytes and neutrophils to inflammatory areas by secreting inflammatory cytokines and chemokines, promoting the inflammatory response (97).

T cells also play important roles in DCM. CD4+ T helper cells (Th1, Th2, Th17 subsets) and CD8 + cytotoxic T cells participate in myocardial inflammation and injury (97). Studies show that the number of regulatory T cells (Treg) in peripheral blood of DCM patients is reduced, impairing their ability to suppress inflammatory responses (98). B cells in DCM can secrete various cytokines, including pro-inflammatory TNF-α and anti-inflammatory IL-1; B cells from patients secrete more TNF-α and less IL-1, indicating impaired anti-inflammatory capacity (97).

Natural killer cells have anti-inflammatory effects, and their depletion can lead to DCM and heart failure (97). Dendritic cells, as antigen-presenting cells, play key roles in initiating and regulating adaptive immune responses.

#### 3.2.3 Cytokine network

Cytokine networks orchestrate the inflammatory response in DCM. Multiple cytokines participate in this network, including IL-1β, IL-6, IL-17A, IL-33, and transforming growth factor-*β*1 (TGF-*β*1). These cytokines can directly cause tissue damage and also promote fibrosis by inducing fibroblast proliferation and collagen deposition (99, 100). IL-6 signaling plays a central role. IL-6, produced by activated macrophages and T cells, promotes differentiation of Th17 cells, enhances B-cell antibody production, and induces acute-phase proteins. Elevated IL-6 levels in DCM correlate with disease severity and adverse outcomes (101). TNF-α is another key pro-inflammatory cytokine that can directly inhibit myocardial contractility, induce cardiomyocyte apoptosis, promote myocardial remodeling, and activate the NF-*κ*B pathway (98). Adaptive immune dysregulation is also involved. DCM patients show increased Th17/Treg ratios and elevated production of pro-inflammatory cytokines by B cells. Autoreactive T cells and B cells produce antibodies against cardiac antigens, perpetuating inflammation.

Notably, some cytokines may have protective effects in DCM. For example, IL-33 can exert anti-hypertrophic and anti-fibrotic effects through the ST2 receptor (102). Soluble ST2 acts as a decoy receptor, neutralizing the protective effects of IL-33, and elevated levels are associated with poor prognosis in DCM (103).

Regarding myocarditis-associated DCM progression, recent evidence suggests that up to 46% of acute myocarditis cases may have an underlying genetic predisposition. Genetic variants in *DSP*, *FLNC*, and *LMNA* have been identified in patients presenting with acute myocarditis who subsequently developed DCM. These findings support the “genetic susceptibility+inflammatory trigger” model of DCM pathogenesis.

### 3.3 Clinical assessment and therapeutic implications of inflammation

#### 3.3.1 Clinical assessment of inflammation

Clinical assessment of myocardial inflammation requires a multimodal approach. Cardiac magnetic resonance is currently the best non-invasive modality for assessing myocardial inflammation; T2-weighted imaging can detect myocardial edema (indicating active inflammation), T1 mapping and extracellular volume can quantitatively assess diffuse myocardial fibrosis, and late gadolinium enhancement can detect focal fibrosis or scar (104). For children with suspected inflammatory DCM, cardiac magnetic resonance should be performed within six months of disease onset to improve diagnostic accuracy (105).

Endomyocardial biopsy remains the gold standard for diagnosing myocarditis, but its use is limited by invasiveness and operator expertise requirements. According to expert consensus, endomyocardial biopsy is indicated for: (1) peripheral blood eosinophilia; (2) persistently or recurrently elevated creatine kinase-MB or troponin levels, especially in the presence of suspected or confirmed autoimmune disease; (3) suspected specific etiology or etiologic diagnosis before major treatment decisions (4); idiopathic DCM to determine whether active myocarditis is present (106–108).

Anti-myocardial antibody testing aids in the etiologic diagnosis of inflammatory DCM. The positive rates of anti-myocardial antibodies in familial and idiopathic DCM are 60% and 41%–85%, respectively (109). DCM patients with evidence of inflammatory lesions, a history of progression from myocarditis to cardiomyopathy, or persistent enterovirus RNA expression have higher anti-myocardial antibody positivity rates (109).

#### 3.3.2 Anti-inflammatory therapy

Given the important role of inflammation in DCM pathogenesis, anti-inflammatory therapy represents a potential treatment strategy. However, current evidence for anti-inflammatory therapy in DCM remains limited.

For children with DCM who have evidence of definite myocarditis or immune injury within six months of disease onset (positive anti-myocardial antibodies or anti-nuclear antibodies, significant cytokine abnormalities, etc.), corticosteroids and/or intravenous immunoglobulin may be considered (110–112). The recommended regimen for prednisone is 1.0–1.5 mg/(kg·day), tapered gradually after 4–12 weeks, with a total course of at least 6 months; intravenous immunoglobulin is given as an initial dose of 2 g/kg in single or divided doses, followed by additional doses at 3 and 6 months (110–112).

However, the overall efficacy of anti-inflammatory therapy remains controversial. Some studies show that immunosuppressive therapy can improve left ventricular function in patients with virus-negative myocarditis, but its effect on survival remains uncertain (110, 113). Currently, anti-inflammatory therapy is not incorporated into routine DCM management guidelines and requires further evidence from multicenter clinical trials (107).

In recent years, novel anti-inflammatory drugs targeting specific inflammatory pathways, such as the IL-1β antagonist anakinra and the IL-6 receptor antagonist tocilizumab, have shown potential in heart failure treatment (114, 115). Their application in DCM warrants further exploration.

## 4 Metabolic reprogramming: the energy crisis of the failing heart

The failing heart is an energy-starved heart. Metabolic reprogramming is a core feature of heart failure progression, involving substrate utilization shifts, mitochondrial dysfunction, and energy depletion (18, 19). This section systematically discusses the manifestations, mechanisms, and clinical implications of metabolic disturbances in DCM.

### 4.1 Mitochondrial dysfunction

#### 4.1.1 Central role of mitochondria in myocardial energy metabolism

Mitochondria are the powerhouses of cardiomyocytes, producing most of the ATP required for sustained myocardial contraction. In the healthy heart, approximately 70% of ATP is derived from fatty acid *β*-oxidation, with the remainder from glucose, lactate, and ketone oxidation (116). Mitochondrial dysfunction is a key mechanism in the progression from DCM to heart failure (19, 117).

#### 4.1.2 Mitochondrial abnormalities in DCM

Multiple mitochondrial abnormalities are observed in myocardial tissue from DCM patients, including: (1) structural changes such as mitochondrial swelling and cristae disruption; (2) reduced activity of mitochondrial electron transport chain complexes; (3) decreased mitochondrial DNA copy number and accumulation of mutations; (4) imbalance in mitochondrial dynamics (fusion and fission abnormalities); (5) abnormal mitophagy (18, 19, 118). Autophagy and mitophagy—the selective degradation of damaged mitochondria—are critical for mitochondrial quality control. Dysregulated mitophagy has been implicated in DCM progression, with both insufficient clearance of damaged mitochondria and excessive degradation of functional mitochondria contributing to energy crisis.

Mitochondrial dysfunction leads to reduced ATP synthesis and increased reactive oxygen species production, with oxidative stress pathways becoming activated, causing lipid peroxidation, protein oxidation, and DNA damage, further impairing mitochondrial function. This creates a vicious cycle, further damaging mitochondrial DNA and cardiomyocyte proteins (117). Studies show that mitochondrial dysfunction correlates with reduced left ventricular ejection fraction and severity of heart failure (118). These findings are primarily derived from adult studies and animal models; pediatric-specific data on autophagy, mitophagy, and oxidative stress pathways in DCM remain limited.

#### 4.1.3 Genetic background and mitochondrial function

Genetic factors can directly contribute to mitochondrial dysfunction. *LMNA* mutations are associated with altered mitochondrial function, and structural abnormalities and energy metabolism disturbances in mitochondria have been observed in myocardial tissue from *LMNA*-deficient mice (34, 118). *TTN* truncating variants can also affect mitochondrial localization and energetics, with reduced mitochondrial ATP production in cardiomyocytes carrying TTNtv (119).

Mitochondrial DNA mutations represent another important cause of pediatric DCM. Barth syndrome (caused by *TAZ* gene mutations) is a classic mitochondrial disease with DCM phenotype, characterized by infantile-onset DCM, neutropenia, skeletal myopathy, and growth retardation (120). Other mitochondrial DNA mutations (such as mitochondrial tRNA gene mutations) can also cause pediatric DCM (121).

### 4.2 Substrate utilization shift and energy metabolic reprogramming

#### 4.2.1 Suppression of fatty acid oxidation

In the failing heart, expression of key regulators of fatty acid oxidation (such as peroxisome proliferator-activated receptor *α*, peroxisome proliferator-activated receptor *γ* coactivator 1*α*) is downregulated, and fatty acid oxidation enzyme activities are reduced, leading to decreased capacity for fatty acid oxidation (116). This downregulation may represent a compensatory response to reduce oxygen demand during fatty acid oxidation, but it also reduces ATP yield per unit of oxygen consumed (116).

#### 4.2.2 Relative enhancement of glucose oxidation

Corresponding to the suppression of fatty acid oxidation, glucose uptake and glycolysis are enhanced in the failing heart, but glucose oxidation does not increase proportionally, leading to uncoupling between glycolysis and glucose oxidation (122). This metabolic remodeling results in more pyruvate being converted to lactate rather than entering the tricarboxylic acid cycle for oxidation, further reducing energy production efficiency (122).

#### 4.2.3 Ketone body metabolism

In recent years, the role of ketone bodies in heart failure metabolic remodeling has gained attention. Ketone bodies are metabolic intermediates produced in the liver during fatty acid oxidation and can serve as alternative energy sources for the heart. Studies show elevated plasma ketone body levels in heart failure patients, possibly representing a compensatory response to energy deficiency (123). Exogenous ketone body supplementation has shown improved cardiac function in heart failure animal models, but clinical application—particularly in the pediatric population—requires further evidence (124).

### 4.3 Insulin resistance

Insulin resistance is a common comorbidity of heart failure, referring to reduced responsiveness of body cells to insulin, leading to impaired glucose uptake and utilization (125). In children with DCM, the prevalence of insulin resistance is higher than in healthy children, particularly in those who are pubertal or obese (121, 126).

The mechanisms linking insulin resistance to heart failure are complex, involving: (1) neurohormonal activation (activation of the sympathetic nervous system and renin-angiotensin-aldosterone system); (2) interference with insulin signaling by inflammatory factors (such as TNF-α, IL-6); (3) reduced skeletal muscle glucose uptake due to decreased peripheral blood flow; (4) enhanced oxidative stress (17, 125).

Insulin resistance further exacerbates myocardial energy crisis, reducing glucose oxidation efficiency and promoting fatty acid accumulation and lipotoxicity (121). Sodium-glucose cotransporter 2 inhibitors (SGLT2i) represent a major breakthrough in heart failure treatment in recent years, with their cardioprotective effects possibly related to improved energy metabolism and reduced insulin resistance (127). Currently, the application of SGLT2i in pediatric heart failure is still in early stages, with preliminary studies showing good tolerability in children (126, 128).

### 4.4 Iron metabolism and myocardial energetics

Iron is a critical cofactor for mitochondrial respiratory chain complexes (I, II, III, IV) and various oxidoreductases, essential for myocardial energy metabolism (129). Iron deficiency is a common comorbidity of heart failure, affecting approximately 40% of chronic heart failure patients, and is associated with reduced exercise capacity, decreased quality of life, and poor prognosis (129, 130).

Iron deficiency is also common in pediatric DCM. Studies show that iron deficiency is associated with more severe heart failure status, higher hospitalization risk, and worse prognosis (131, 132). Iron deficiency impairs mitochondrial respiratory chain function, reduces ATP synthesis, and increases reactive oxygen species production, exacerbating myocardial energy crisis (129).

Intravenous iron supplementation is an effective strategy for improving symptoms and outcomes in heart failure patients with iron deficiency. Randomized clinical trials and meta-analyses show that intravenous iron (ferric carboxymaltose, ferric derisomaltose) reduces heart failure hospitalization risk, though it does not significantly affect all-cause mortality (133–135). Intravenous iron is now recommended by guidelines for HFrEF patients with iron deficiency (7). In pediatric DCM, iron supplementation should also be considered, especially for those with iron deficiency (136).

### 4.5 Electrolyte disturbances

Electrolyte disturbances are common in children with DCM, especially those receiving diuretic therapy. Disturbances in potassium, magnesium, sodium, and calcium not only affect myocardial electrical stability but also participate in myocardial energy metabolism regulation.

Hypokalemia is one of the most common electrolyte disturbances in children with DCM. Loop diuretics and thiazide diuretics increase renal potassium excretion, leading to hypokalemia (137). Hypokalemia increases the risk of ventricular arrhythmias and sudden cardiac death and may reduce the efficacy of some heart failure medications (such as digoxin) (138). Studies show that maintaining serum potassium at 4.0–5.0 mmol/L significantly reduces the risk of ventricular tachycardia and sudden cardiac death (139).

Hypomagnesemia often coexists with hypokalemia and can lead to malignant arrhythmias such as torsade de pointes (138). When serum magnesium is <0.5 mmol/L or torsade de pointes occurs, urgent intravenous magnesium supplementation is indicated (140).

Hyponatremia is a marker of poor prognosis in children with DCM, often resulting from diuretic use, neurohormonal activation, and fluid retention (138). The key to managing hyponatremia is to address underlying causes and correct coexisting hypokalemia and hypomagnesemia; simple sodium supplementation is not recommended (141).

### 4.6 Metabolic intervention strategies

Based on the important role of metabolic reprogramming in heart failure progression in DCM, metabolic interventions represent potential treatment strategies.

Myocardial metabolic modulators are commonly used as adjunctive therapies, including coenzyme Q10, levocarnitine, creatine phosphate, and fructose-1,6-diphosphate (142–144). Coenzyme Q10 is a component of the mitochondrial electron transport chain involved in ATP synthesis; levocarnitine facilitates entry of long-chain fatty acids into mitochondria for *β*-oxidation. Available evidence suggests these agents may improve myocardial energy metabolism and cardiac function, though high-quality randomized controlled trials are lacking.

SGLT2 inhibitors (dapagliflozin, empagliflozin) are first-line therapies for HFrEF in adults, significantly reducing the risk of heart failure hospitalization and cardiovascular death (127, 145). Their cardioprotective mechanisms involve multiple aspects, including improved myocardial energy metabolism, reduced cardiac load, inhibition of fibrosis, and improved mitochondrial function (146). In pediatric DCM, SGLT2i application is still in early stages. Early experience suggests SGLT2i are well tolerated in children and may improve left ventricular function and quality of life, particularly in those with puberty or insulin resistance (126, 128). Currently, SGLT2i are not approved for children and require further clinical trial evidence.

Iron supplementation has become standard therapy for heart failure patients with iron deficiency (7). Intravenous iron (ferric carboxymaltose, ferric derisomaltose) improves myocardial energy metabolism, exercise capacity, and quality of life, and reduces heart failure hospitalization risk (133, 134). In pediatric DCM, iron deficiency should be actively treated to reduce heart failure and adverse event rates (131, 136).

## 5 Integrating the “gene-inflammation-metabolism” axis: toward precision medicine

The complexity of pediatric DCM lies in the interaction among genetics, inflammation, and metabolism. This section integrates the preceding content, proposes a unified model for understanding disease progression, and explores precision medicine strategies based on this model.

### 5.1 A unified model: from genetic susceptibility to heart failure

**Figure 1:**
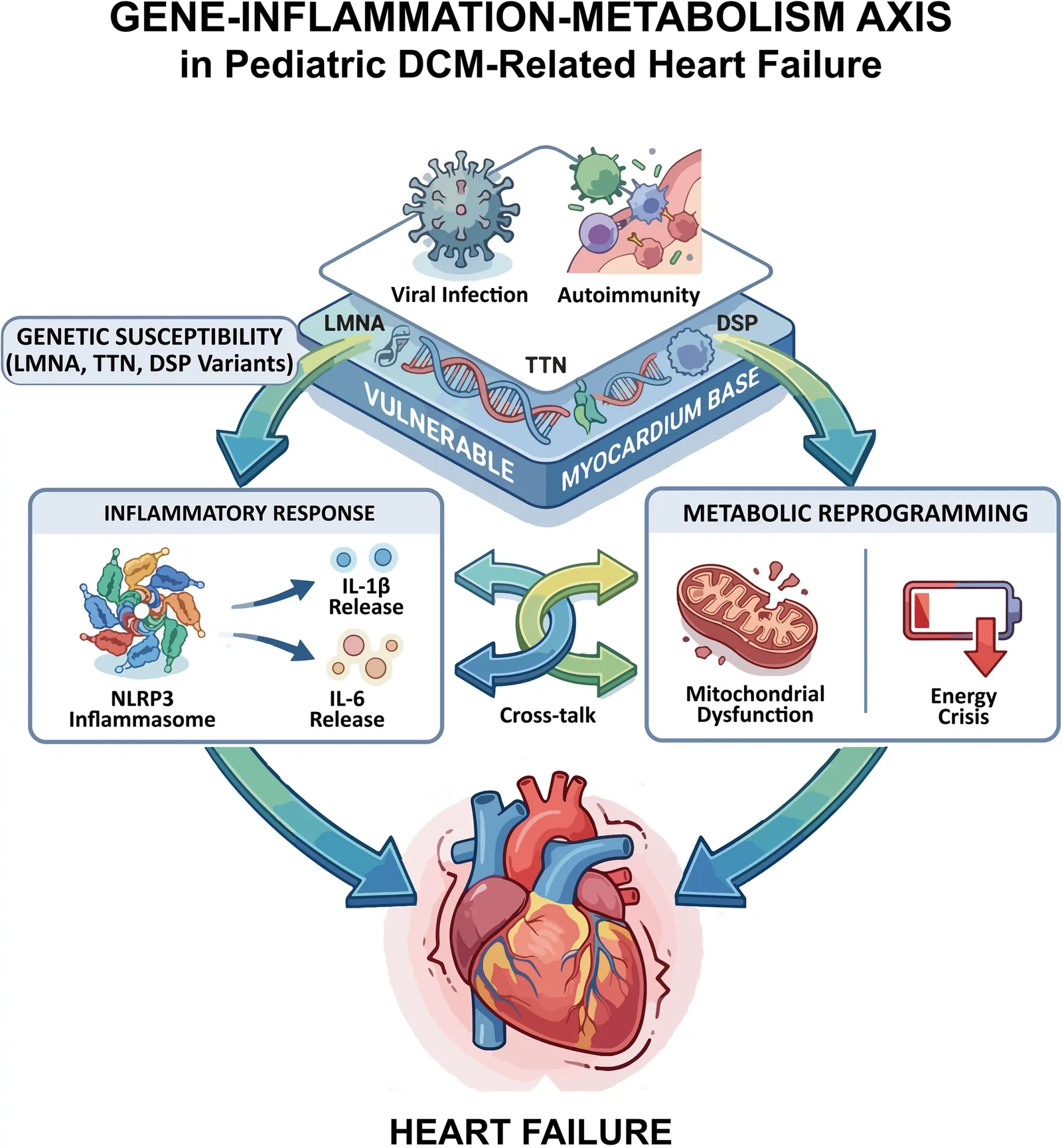
Integrated model of heart failure progression in pediatric dilated cardiomyopathy. Schematic illustration of the “gene-inflammation-metabolism” axis driving heart failure progression in pediatric dilated cardiomyopathy. Genetic susceptibility (e.g., *LMNA*, *TTN* truncating variants, *DSP* variants) creates a vulnerable myocardial substrate. Environmental triggers (viral infection, autoimmunity, metabolic stress) initiate the inflammatory response via inflammasome activation and cytokine release. This inflammatory response directly damages the myocardium and, through fibroblast activation and collagen deposition, leads to fibrosis and ventricular remodeling. Concurrently, genetic defects and inflammation jointly drive metabolic reprogramming, including mitochondrial dysfunction, substrate utilization shifts, and insulin resistance, which exacerbate the energy crisis. The interplay of these three domains ultimately results in progressive heart failure.

Based on available evidence, we propose an integrated model of heart failure progression in pediatric DCM (Figure 1). This model includes three core components and multiple interactive processes:

1. Genetic susceptibility as the foundation: Children carrying pathogenic gene variants (such as TTN truncating variants, LMNA mutations, DSP mutations) have congenital defects in cardiomyocyte structure and function, forming a vulnerable myocardial substrate. These defects may include sarcomere structural abnormalities, nuclear envelope instability, and intercellular junction dysfunction.
2. Environmental triggers as catalysts: Against a background of genetic susceptibility, environmental triggers (such as viral infection, autoimmune response, metabolic stress) can activate the inflammatory response. These triggers activate Toll-like receptors and the NLRP3 inflammasome through damage-associated molecular patterns and pathogen-associated molecular patterns, initiating the IL-1 cascade.
3. Inflammatory response as an amplifier: The inflammatory response is initially a defensive mechanism but can transform into maladaptive chronic inflammation in the context of genetic susceptibility. Inflammatory cytokines (IL-1β, IL-6, TNF-α) not only directly damage cardiomyocytes but also promote collagen deposition and fibrosis by activating fibroblasts, leading to ventricular remodeling.
4. Metabolic reprogramming as the final pathway: Inflammatory responses and genetic defects together lead to mitochondrial dysfunction and substrate utilization shifts, shifting myocardial energy metabolism from efficient fatty acid oxidation to inefficient glycolysis, resulting in reduced ATP synthesis and increased reactive oxygen species production, ultimately causing systolic dysfunction and heart failure.

These three components do not interact in a simple linear fashion but form a complex interactive network. For example, genetic background can influence the intensity of inflammatory responses (as seen in *DSP* and *FLNC* variant-associated “hot phase” myocarditis); inflammatory factors can further inhibit mitochondrial function (such as TNF-α inhibition of respiratory chain complex activity); metabolic disturbances can in turn enhance inflammatory responses (such as reactive oxygen species activation of the NLRP3 inflammasome).

### 5.2 Precision medicine strategies based on the “gene-inflammation-metabolism” axis

#### 5.2.1 Precision risk stratification

Traditional risk stratification relies primarily on left ventricular ejection fraction, New York Heart Association functional classification, and other clinical indicators, which inadequately capture the heterogeneity of DCM. Multi-dimensional risk stratification based on the “gene-inflammation-metabolism” axis promises improved predictive accuracy.

Genotype is central to risk stratification. As discussed, *LMNA*, *FLNC*, *DSP*, *RBM20*, and *PLN* variants are associated with high risk of malignant arrhythmias and sudden cardiac death, warranting enhanced monitoring and consideration of implantable cardioverter-defibrillator implantation for primary prevention (36, 37, 51, 56, 60). Patients carrying *TTN* variants have higher LVRR rates and may respond better to medical therapy (31, 72).

Imaging biomarkers are important supplements to risk stratification. Cardiac magnetic resonance is the gold standard for assessing myocardial fibrosis, and the presence and extent of late gadolinium enhancement correlate with arrhythmic risk (147). A ring-like late gadolinium enhancement pattern is characteristic of *FLNC* and *DSP*-associated DCM, indicating high arrhythmic risk (51, 54). Global longitudinal strain and other echocardiographic parameters can detect subclinical systolic dysfunction early and correlate with poor prognosis (74, 148).

Inflammatory and metabolic biomarkers are emerging tools for risk stratification. High-sensitivity C-reactive protein (hs-CRP) and IL-6 levels are elevated in DCM patients and correlate with worse outcomes, reflecting systemic inflammation (98), though these markers lack specificity for myocardial inflammation. Alongside these inflammatory markers, NT-proBNP is the most established biomarker for heart failure severity and prognosis assessment in both children and adults (149), and serial measurements can monitor treatment response and predict clinical deterioration. Cardiac troponins (cTnI and cTnT) are markers of myocardial injury; in DCM, troponin elevation may indicate ongoing myocardial damage, inflammation, or microvascular dysfunction, and persistent elevation in the absence of acute coronary syndrome suggests active myocardial injury and portends a worse prognosis. Novel fibrosis-related biomarkers have also shown promise: galectin-3 (Gal-3) is a mediator of cardiac fibrosis and inflammation, with elevated levels associated with adverse remodeling and heart failure progression, while soluble ST2 (sST2) is a decoy receptor for IL-33 that neutralizes its cardioprotective effects and is a powerful predictor of mortality in heart failure, reflecting both fibrotic and inflammatory processes; however, pediatric-specific data for these biomarkers remain limited. Circulating microRNAs are emerging as potential non-invasive biomarkers, with miR-1, miR-133a, and miR-208 implicated in cardiac hypertrophy, fibrosis, and apoptosis, and while microRNA signatures may differentiate DCM subtypes and predict therapeutic responses, clinical translation in pediatrics is still in its infancy. Beyond these emerging circulating biomarkers, routine metabolic markers also carry prognostic value: abnormal iron metabolism markers (serum iron, ferritin, transferrin saturation) are associated with reduced exercise capacity and poor prognosis (131, 132). Metabolomic biomarkers are also gaining attention, as specific metabolic profiles—such as alterations in amino acid, lipid, and energy metabolism pathways—have been associated with heart failure severity and outcomes (150), and metabolomic profiling may reveal novel pathways for therapeutic targeting and improve risk stratification, though the high cost and complexity of metabolomic analyses currently limit their clinical application. While these biomarkers show promise, several limitations exist: most biomarker studies are small, cross-sectional, and derived from adult cohorts; biomarker levels can be influenced by age, renal function, comorbidities, and medications; and the incremental value of novel biomarkers over established markers such as NT-proBNP and LVEF remains uncertain. Future studies should therefore focus on establishing pediatric-specific reference ranges, standardizing assay methods, and developing multi-marker models that combine biomarkers from different pathophysiological pathways.

#### 5.2.2 Targeted therapies

Targeted therapies based on the “gene-inflammation-metabolism” axis are at the core of precision medicine for DCM.

Targeting genetic defects represents the ultimate precision therapy. Gene therapy is a current research hotspot, encompassing three main strategies: gene replacement therapy, which introduces a functional copy of the defective gene; gene silencing, which suppresses expression of mutant alleles; and genome editing, which directly corrects the pathogenic mutation (151, 152).

Adeno-associated virus (AAV)-mediated gene replacement is the most widely explored approach. AAV vectors offer favorable safety profiles and efficient cardiomyocyte transduction. However, their packaging capacity is limited to genes <5 kb, which precludes delivery of larger genes such as *TTN* (∼100 kb). AAV9-based gene replacement therapy has entered clinical trials for Danon disease (*LAMP2* mutations) and PKP2-associated arrhythmogenic cardiomyopathy, with promising early results (120, 153). For DCM-associated genes such as *BAG3*, *LMNA*, and *RBM20*, several gene replacement trials are ongoing or in preclinical development (40, 154, 155).

CRISPR-Cas9 genome editing offers the potential for permanent correction of pathogenic mutations. Recent studies in animal models have successfully corrected *MYH7* and *RBM20* mutations, preventing cardiomyopathy development (155, 156). Base editing and prime editing technologies further enhance precision and safety by enabling single-nucleotide corrections without double-strand breaks.

RNA-based therapies represent an emerging alternative. Antisense oligonucleotides can modulate splicing to correct *RBM20*-related splicing defects. Small interfering RNA (siRNA) and microRNA-based approaches are also under investigation for gene silencing.

Challenges specific to pediatric patients include: the need for long-term safety data given the prolonged lifespan of children; the potential impact of gene therapy on cardiac development and growth; the risk of immune responses to viral vectors, which may be more pronounced in children; the optimal timing of intervention—whether gene therapy should be administered before or after disease manifestation; and the high cost and limited accessibility of these therapies. Addressing these challenges will require dedicated pediatric clinical trials and long-term follow-up registries.

Targeting inflammation is applicable to DCM patients with evidence of active inflammation. Immunosuppressive therapy (corticosteroids, azathioprine, intravenous immunoglobulin) may be used for myocarditis-associated DCM (110–112). Targeted agents such as the IL-1β antagonist anakinra and IL-6 receptor antagonist tocilizumab have shown potential in myocarditis and heart failure (114, 115). Specific inhibitors of the NLRP3 inflammasome are also under development.

Targeting metabolism represents a relatively mature treatment strategy. SGLT2 inhibitors are first-line therapy for HFrEF in adults and have promising applications in pediatric DCM (127, 145). For children with DCM who are pubertal or have insulin resistance, SGLT2 inhibitors may be considered (126, 128). Intravenous iron supplementation is indicated for DCM children with iron deficiency to improve myocardial energy metabolism and cardiac function (133, 134). Myocardial metabolic modulators (coenzyme Q10, levocarnitine, etc.) can be used as adjunctive therapies (142–144).

**Figure 2:**
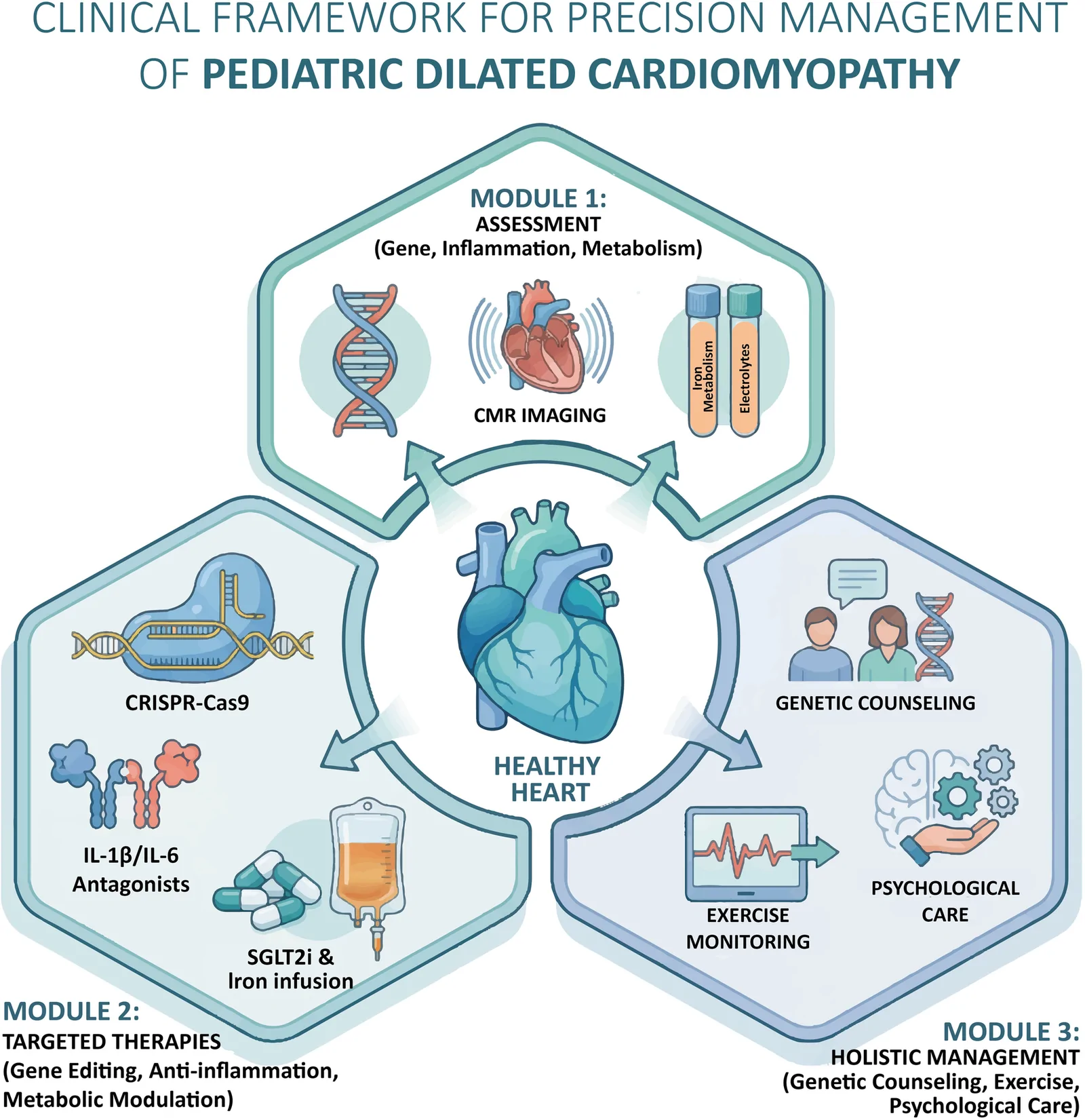
Clinical framework for precision management of pediatric dilated cardiomyopathy based on the “Gene-Inflammation-Metabolism” axis. Proposed clinical workflow integrating the “gene-inflammation-metabolism” axis for precision management of pediatric dilated cardiomyopathy (DCM). Following initial DCM diagnosis, comprehensive assessment is performed, comprising: (1) genetic assessment (genetic testing, family screening); (2) inflammation assessment (cardiac magnetic resonance, endomyocardial biopsy, anti-myocardial antibodies, inflammatory markers); and (3) metabolic assessment (iron metabolism, electrolytes, insulin resistance). These results are then used for risk stratification and to identify actionable therapeutic targets, enabling individualized therapy: targeting genetics (gene therapy, genotype-guided drug selection), targeting inflammation (immunosuppression, anti-inflammatory therapy), and targeting metabolism (SGLT2 inhibitors, iron supplementation, metabolic modulators). Dynamic reassessment during follow-up allows adjustment of treatment strategies based on disease evolution.

Figure 2 presents a clinical framework for precision management of DCM based on the “gene-inflammation-metabolism” axis.

#### 5.2.3 Monitoring and management

Monitoring and management based on the “gene-inflammation-metabolism” axis should be integrated throughout the disease course of children with DCM.

Genetic counseling is an important component of DCM management. For probands diagnosed with DCM, three-generation family history investigation and cardiac screening of first-degree relatives should be performed (73, 105). If a pathogenic gene variant is identified in the proband, children in the family should undergo clinical screening annually; if genetic testing in the proband is negative, children in the family should undergo clinical screening every 3–5 years (157). For families with identified pathogenic variants, preconception genetic counseling and prenatal diagnosis options should be provided.

Lifestyle management is crucial for children with DCM. Moderate aerobic exercise can improve cardiac function and quality of life, but exercise intensity should be individually assessed (158). Children with stage C and D DCM should avoid competitive sports and moderate-to-high-intensity physical activity. Maintaining healthy weight, balanced nutrition, and infection prevention are basic management measures.

Psychological support should not be overlooked. Children with DCM and their families often experience anxiety, depression, adjustment disorders, and other emotional problems that should be promptly assessed and managed with psychological support and counseling (159).

#### 5.2.4 Psychological stress as a modifiable “second hit”

Chronic psychological stress is increasingly recognized as a biologically relevant amplifier of disease progression in pediatric DCM, acting as a modifiable environmental “Second Hit” that sustains the inflammatory-metabolic vicious cycle. Children with DCM and their families face unique psychological challenges, including anxiety about disease progression, fear of sudden death, limitations on physical activity, frequent hospitalizations, and uncertainty about long-term outcomes.

Psychobiological pathways linking stress to disease progression: Chronic psychological distress activates the hypothalamic-pituitary-adrenal (HPA) axis and the sympathetic nervous system, directly elevating circulating inflammatory cytokines such as IL-6 and TNF-α and worsening insulin resistance. This sustained neurohormonal activation feeds back into both the inflammatory and metabolic arms of the “gene-inflammation-metabolism” axis, creating a self-perpetuating vicious cycle. In a genetically susceptible child, psychological stress may act as an additional “hit” that exacerbates myocardial inflammation and metabolic dysfunction.

Pediatric-specific psychological interventions: Psychological support should be an integral component of multidisciplinary care. Evidence-based approaches with demonstrated benefits in pediatric chronic illness include: (1) Cognitive-behavioral therapy (CBT): Helps children reframe negative thoughts, develop coping strategies, and reduce disease-related anxiety. CBT has been shown to improve quality of life and reduce stress-related symptoms in pediatric cardiac patients. (2) Relaxation techniques and mindfulness meditation: Deep breathing, guided imagery, and mindfulness practices can reduce sympathetic activation, lower cortisol levels, and improve emotional regulation. (3) Hypnotherapy and regression therapy: May be beneficial for children experiencing procedure-related anxiety or trauma, though pediatric cardiac-specific data are limited. (4) Family-based interventions: Address the psychological burden on caregivers, who often experience high rates of anxiety, depression, and post-traumatic stress symptoms that can indirectly affect the child's psychological well-being.

Integration into clinical practice: Regular psychological screening (using validated pediatric tools such as the Pediatric Quality of Life Inventory or Screen for Child Anxiety Related Emotional Disorders) should be performed during follow-up visits. Referral to child psychology or psychiatry services should be offered when indicated. Psychological support should be seen not as an optional “soft” add-on, but as a core component of disease management that can modulate the inflammatory and metabolic drivers of disease progression.

#### 5.2.5 Artificial intelligence and multi-omics integration

The integration of artificial intelligence (AI) and machine learning (ML) with multi-omics data holds transformative potential for precision medicine in pediatric DCM. AI algorithms can analyze large datasets combining genomic, transcriptomic, proteomic, metabolomic, and imaging data to identify novel disease subtypes, predict disease trajectories, and guide treatment decisions.

Machine learning applications in DCM include: automated interpretation of echocardiographic and cardiac magnetic resonance imaging for early detection of subclinical dysfunction; integration of genotype, clinical, and biomarker data for improved risk stratification; identification of novel biomarkers through unsupervised learning; and prediction of treatment response using supervised models.

Multi-omics integration offers a comprehensive view of disease pathogenesis. Genomics identifies causal variants; transcriptomics reveals gene expression changes; proteomics captures protein-level alterations; and metabolomics reflects downstream metabolic consequences. Integrating these layers can identify key drivers of disease progression and reveal novel therapeutic targets.

Predictive modeling for pediatric heart failure outcomes is a promising application. Models that combine genotype, imaging, biomarkers, and clinical parameters can estimate individual risk of death, transplantation, or major adverse cardiac events. However, pediatric-specific models are scarce, and most existing tools have been developed in adult populations. Large collaborative consortia and shared datasets are needed to develop and validate pediatric-specific predictive models.

### 5.3 Critical appraisal and controversies

Despite the conceptual appeal of the “gene-inflammation-metabolism” axis, several controversies and limitations warrant critical appraisal. First, in genetic studies, while TTNtv are consistently the most common genetic cause of DCM, their high prevalence in healthy individuals (∼1%–3%) raises questions about variant interpretation and penetrance. Not all TTNtv are pathogenic; variants located in constitutively expressed exons (such as the A-band) have higher pathogenicity than those in poorly expressed regions, highlighting the need for improved variant classification frameworks and functional validation. Second, regarding inflammatory biomarkers, although elevated hs-CRP, IL-6, and TNF-α levels correlate with adverse outcomes, their specificity for DCM is limited. Anti-heart autoantibodies are detected in up to 60% of DCM patients, but their pathogenic role vs. epiphenomenon status remains debated, and the optimal threshold for immunotherapy is undefined, with immunosuppressive therapy failing to demonstrate consistent survival benefits across studies. Third, genotype-based risk prediction, while advanced by genotype-phenotype correlations, is not absolute; variable penetrance and expressivity within the same genotype family challenge the reliability of such predictions. Many risk scores (e.g., LMNA risk score, PLN risk calculator) were developed in adult cohorts and require pediatric validation, and the clinical significance of variants of uncertain significance (VUS) poses a major challenge for clinicians and families. Finally, the translational gap between molecular discoveries and clinical practice remains wide: gene therapy faces vector safety, delivery efficiency, and long-term durability challenges; metabolic modulators such as SGLT2 inhibitors have shown efficacy in adults but lack pediatric approval and dosing data; and the heterogeneous nature of DCM necessitates large collaborative registries to generate sufficient evidence for subgroup-specific recommendations, while the cost of genetic testing and emerging therapies limits accessibility in resource-limited settings.

## 6 Future perspectives and directions

Despite significant advances in pediatric DCM research in recent years, many questions remain unanswered. Based on the “gene-inflammation-metabolism” axis, future research should focus on the following directions:

1. Elucidate developmental stage-specific mechanisms: The age of onset of pediatric DCM spans from infancy to adolescence, with significant differences in metabolic characteristics, immune status, and gene expression across developmental stages. Future studies should systematically compare the pathological mechanisms of DCM across different age groups to provide a basis for age-specific treatment strategies.
2. Develop novel biomarkers: Current risk stratification for DCM still relies heavily on traditional indicators. Novel biomarkers based on multi-omics approaches (genomics, transcriptomics, proteomics, metabolomics) promise to improve prognostic prediction accuracy and guide individualized therapy.
3. Conduct pediatric-specific clinical trials: SGLT2 inhibitors, intravenous iron, and anti-inflammatory agents have shown good efficacy in adult DCM but have limited evidence in children. Well-designed, adequately powered pediatric-specific clinical trials are urgently needed to assess the safety, efficacy, and appropriate dosing of these promising therapies.
4. Advance clinical translation of gene therapy: Gene therapy is the ultimate goal of precision medicine for DCM. Current gene therapy applications in DCM are still in early stages, requiring solutions to key issues such as vector safety, targeting specificity, and immunogenicity, with priority given to genes with the worst prognosis (such as *LMNA*, *FLNC*, *RBM20*).
5. Establish multicenter collaborative networks: Pediatric DCM is a rare disease, making it difficult to obtain sufficient sample sizes from single-center studies. Establishing multicenter, international collaborative networks (such as the DCM SHaRe Registry) to integrate clinical and genetic data is essential for advancing DCM research (36).
6. Leverage single-cell and spatial transcriptomics for molecular characterization: Single-cell sequencing and spatial transcriptomics represent the frontier of molecular characterization. Single-cell RNA sequencing can delineate cellular heterogeneity within the failing heart, identifying distinct cardiomyocyte subtypes, immune cell populations, and fibroblast activation states. Spatial transcriptomics adds a geographical dimension, revealing how gene expression varies across different myocardial regions and how local microenvironments drive disease progression. Pediatric-specific applications of these technologies are still emerging, but they hold promise for understanding age-specific pathological mechanisms.
7. Integrate digital health monitoring and wearable technologies for continuous care: Digital health monitoring and wearable technologies offer opportunities for continuous, non-invasive monitoring of children with DCM. Wearable devices can track heart rate, physical activity, sleep patterns, and ECG rhythms. Integration with AI-powered analytics can provide early warning of clinical deterioration, reduce hospitalizations, and enable proactive management. Pediatric-specific considerations include device miniaturization, user-friendliness for children and caregivers, and data privacy concerns.

## 7 Conclusions

Pediatric dilated cardiomyopathy is a complex, heterogeneous disease whose progression to heart failure is driven by dynamic interactions between genetic susceptibility, inflammatory responses, and metabolic reprogramming. The principal novelty of this review lies in its proposal of a pediatric-specific “gene-inflammation-metabolism” triaxial framework that unifies diverse pathogenic pathways into a clinically actionable model for risk stratification and targeted intervention. This framework integrates genetic susceptibility, inflammatory responses, and metabolic reprogramming to provide a comprehensive understanding of the heterogeneous pathogenesis and clinical outcomes of pediatric DCM. The genetic substrate (particularly key gene variants such as *TTN*, *LMNA*, *FLNC*, *DSP*, *RBM20*) constructs the susceptible myocardium; environmental triggers (viral infection, autoimmunity, etc.) activate inflammatory responses; inflammatory factors and genetic defects together lead to mitochondrial dysfunction, substrate utilization shifts, and energy crisis, ultimately resulting in heart failure.

Precision medicine strategies based on this framework include multi-dimensional risk stratification (integrating genotype, imaging, inflammatory and metabolic biomarkers), targeted therapies (gene therapy, anti-inflammatory therapy, metabolic interventions), and individualized management. Future research should focus on pediatric-specific mechanisms, development of novel biomarkers, pediatric clinical trials, and translation of gene therapy. By integrating basic research with clinical practice, we can hope to provide more precise and effective diagnostic and treatment strategies for children with DCM, improving their quality of life and long-term outcomes.
